# Narcissistic predispositions of self-harm in young women with and without depression

**DOI:** 10.1192/j.eurpsy.2024.1364

**Published:** 2024-08-27

**Authors:** O. U. Vorontsova, T. I. Medvedeva, O. M. Boyko, S. N. Enikolopov, S. O. Kuznetsova

**Affiliations:** Clinical psychology, Federal Stare Budgetary Scientific Institution “Mental Health Research Center”, Moscow, Russian Federation

## Abstract

**Introduction:**

Diagnostic criteria for narcissistic personality disorder primarily focus on grandiosity and significance. In psychotherapeutic work, it is important to distinguish two subtypes of pathological narcissism: narcissistic grandiosity and narcissistic vulnerability. One of the manifestations of narcissistic traits is unstable or unformed self-esteem, manifested in attempts to conform to ideals. A part of modern society perceives the female body as an object that “needs to be looked at”. Self-objectification refers to a learned pattern of self-assessment of the importance of one’s body and appearance compared to other aspects of the self. Self-observation and comparison of oneself with others is one of the manifestations of self-objectification. With acts of auto aggression, the body becomes a tool or a means to solve psychological problems. In order for this to become possible, the ability to objectify your body “to look at it from the outside” plays an important role.

**Objectives:**

Analysis of the relationship between non-suicidal self-injurious behavior and narcissistic personality traits in young women with depression and young women without a psychiatric diagnosis.

**Methods:**

Тhe study included 49 women divided into two groups. The first group included 24 patients with depression undergoing inpatient treatment (mean age 18.4). The second group included 25 healthy subjects (mean age 18 years). The methods: The answer to the question “Sometimes I purposely injure myself” was used as an indicator of self-harm (NSSI) (five-point Likert scale); “Ich structure test” (ISTA); “Physical Appearance Comparison Scale-Revised” (PACS-R).

**Results:**

In the clinical group, a significant association of severity of NSSI with indicators of “deficit narcissism” was revealed (Spearman r=,534*). Correlations were found between the severity of NSSI and PACS-R (r=,344**). In the clinical group, there was no connection between “Comparison with others” and narcissistic traits. In a group of healthy subjects, significant associations of NSSI severity with “destructive narcissism” (,572**) and PACS-R (,576**) were revealed. In the clinical group, the severity of NSSI is associated with a more serious pathology - the lack of formation of “normal” narcissism, and in the healthy group it is more likely to be deformed narcissism. Self-objectification and comparison of oneself with others in the clinical group is not associated with manifestations of narcissistic traits, such connections are demonstrated in the group of healthy young women.

**Conclusions:**

It is shown that in the clinical group of depressed young women, the severity of self-harming behavior is associated with “deficit narcissism”, and in healthy young women, first of all, with “destructive narcissism” with an increased need to compare themselves with others.

**Disclosure of Interest:**

None Declared

